# Integrated Analysis of DEAD-Box Helicase 56: A Potential Oncogene in Osteosarcoma

**DOI:** 10.3389/fbioe.2020.00588

**Published:** 2020-06-26

**Authors:** Chen Zhu, Xianzuo Zhang, Nikolaos Kourkoumelis, Yong Shen, Wei Huang

**Affiliations:** ^1^Division of Life Sciences and Medicine, Department of Orthopedics, The First Affiliated Hospital of USTC, University of Science and Technology of China, Hefei, China; ^2^Department of Medical Physics, School of Health Sciences, University of Ioannina, Ioannina, Greece; ^3^Institute on Aging and Brain Disorders, The First Affiliated Hospital of University of Science and Technology of China, Hefei, China; ^4^Division of Life Sciences and Medicine, Neurodegenerative Disorder Research Center, University of Science and Technology of China, Hefei, China

**Keywords:** biomarker, DEAD-box RNA helicases 56 (DDX56), osteosarcoma, oncogene, proliferation

## Abstract

**Background:** Osteosarcoma is a solid tumor common in the musculoskeletal system. The DEAD-box helicase (DDX) families play an important role in tumor genesis and proliferation.

**Objective:**To screen potential molecular targets in osteosarcoma and elucidate its relationship with DDX56.

**Methods:** We employed the Gene Expression Omnibus and The Cancer Genome Atlas datasets for preliminary screening. DDX56 expression was measured by RT-qPCR in three osteosarcoma cell lines. Biological roles of DDX56 were explored by Gene ontology, Kyoto Encyclopedia of Genes and Genomes and Ingenuity Pathway Analysis. Cell proliferation, cycle, and apoptosis assays were performed using Lentivirus™ knockdown technique.

**Results:** It was found that DDX56 expression was regularly upregulated in osteosarcoma tissue and cell lines, while DDX56 knockdown inhibited cell proliferation and promoted cell apoptosis.

**Conclusions:** The findings suggest DDX56 as a potential therapeutic target for the treatment of osteosarcoma.

## Introduction

Osteosarcoma derives from primitive bone-forming mesenchymal cells. It is a primary bone neoplasm characterized by the production of osteoid or immature bone by the malignant cells (Anderson, 2016). Being the most common primary malignancy of bone in children and adolescents, the incidence of osteosarcoma are 4–5 per year per million for all races and both sexes (Ottaviani and Jaffe, 2009). The survival rate of patients with osteosarcoma has improved mostly due to marked advances in diagnosis and chemotherapy (Anderson, 2016). However, the high rate of relapse and distant metastasis of osteosarcoma result in poor long-term survival (Kumar et al., 2017). Thus, there is an urgent need to develop new treatment strategies for osteosarcoma.

High-throughput microarrays are promising tools for identifying candidate molecular targets in medical oncology. During the last decade, numerous gene expression profiling studies on osteosarcoma oncogenesis and proliferation were performed using microarray technology and showed hundreds of differentially expressed genes (DEGs) involved in different pathways, biological processes, or molecular functions.

The emerging roles of Asp–Glu–Ala–Asp (DEAD)-box RNA helicases have recently been acknowledged in disparate cellular functions. DEAD-box (DDX) RNA helicases play a crucial role not only in unwinding double-stranded RNA molecules but also in transcription, splicing, RNA transport, ribosome biogenesis, RNA editing, RNA decay, and translation (Sugiura et al., 2007; Xu and Hobman, 2012). Although DDX56 is reported to be required in virus infection (Reid and Hobman, 2017), affecting the response to abiotic stress and host–pathogen interaction (Pragya et al., 2007; Umate et al., 2010), the relationship of DDX RNA helicases with malignancies remains unclear. The relationship between DDX family and cancer is worth further investigating since a number of DEAD-box RNA helicases were recently reported to be implicated in solid tumor progression and chemotherapy resistance (Kuramitsu et al., 2013; He et al., 2018).

## Materials and Methods

### Patients and Samples

The gene expression microarray profile of GSE126209 was downloaded from the Gene Expression Omnibus (GEO) database (http://www.ncbi.nlm.nih.gov/geo/). The mRNA profiles of osteosarcoma tumors and adjacent normal tissues were generated by high-throughput sequencing based on the GPL20301 platform (Illumina HiSeq 4000, *Homo sapiens*). Eleven samples were obtained from six Chinese Uyghur patients, in which 10 samples were paired tumor/normal specimens from the same patients. Specifically, gene expression profiles of mesenchymal stem cell osteosarcoma patients were compared with those in non-neoplastic patient to identify the DEGs. The DEAD-box family was given special attention among the DEGs. qPCR was used to examine if DDX56 is highly expressed in osteosarcoma cell lines. Subsequently, we employed the Lentivirus™ technique to examine the effect of DDX56 silencing on human osteosarcoma cell growth *in vitro*.

### Identification of Differentially Expressed mRNAs

Paired *t*-test was used to filter differentially expressed mRNAs between tumor and adjacent normal tissues. We selected differentially expressed genes according to the *p*-value threshold and absolute value of fold change (FC). A value of *p* < 0.05 with |FC| > 2 was considered to represent a significant difference. The Ensembl Gene ID of the mRNAs was transferred into gene symbol using the Biomart module in Ensembl (http://www.ensembl.org/biomart/martview/).

### Hierarchical Clustering

The differentially expressed profiles of mRNAs in DDX 56 family were clustered using a hierarchical cluster algorithm with average linkage and Spearman's rank correlation distance, as provided by the software EPCLUST (http://ep.ebi.ac.uk/EP/EPCLUST/). The clustering was performed using the methods outlined in a previous publication (Misha et al., 2004). Results were visualized with the help of heatmaps and dendrograms.

### Protein–Protein Interaction Network Analysis

The protein–protein interaction (PPI) pairs between differentially expressed mRNAs were identified using the IID (Integrated Interactions Database, version 2018-11) database (Kotlyar et al., 2016), tissue-specific protein–protein interactions (PPIs) with larger information (a total of 1,566,043 PPIs among 68,831 proteins). The PPI interaction in this study was specified in musculoskeletal tissues. Furthermore, Cytoscape (version 3.5.0) was used to establish the PPI network and calculate the parameters of nodes and edges. Top nodes in the DDX56 family net were chosen according to the network topology property indicators, and were analyzed by CytoNCA in Cytoscape for factors including degree, betweenness centrality, and closeness centrality. In general, a high indicator score in network topology denotes an important role in the network. Top nodes with the highest degree were selected for further study.

### mRNA Profile Data and Survival Analysis

The mRNA profile and its corresponding survival data were retrieved from The Cancer Genome Atlas (TCGA) database (https://tcga-data.nci.nih.gov/tcga/). These data were analyzed using the UALCAN (http://ualcan.path.uab.edu/) portal tools (Chandrashekar et al., 2017). The UALCAN tools enable graphs and plots depicting gene expression and patient survival information based on gene expression. Additional information about the selected genes was provided by GTEx (https://gtexportal.org/). Genes positively and negatively correlated with DDX56 in sarcoma (SARC) patients were screened out according to GTEx Profiles (Lonsdale et al., 2013). Extremely low-expressed genes (median TPM < 0.5) were filtered out.

### Osteosarcoma Cell Lines

Human osteosarcoma cell lines (HOS, Sao-2, and U-2 OS) were purchased from the Shanghai Cell Bank (Shanghai, China). Cell lines were cultured in Dulbecco's modified Eagle's medium (DMEM; HyClone, Tauranga, New Zealand) supplemented with 10% fetal bovine serum (FBS; Gibco, Rockville, MD, USA), 100 μg/ml of streptomycin (Sigma-Aldrich, St. Louis, MO, USA), and 100 U/ml of penicillin (Sigma-Aldrich), followed by incubation in a humidified atmosphere with 5% CO_2_ at room temperature.

### Functional Enrichment Analysis

Gene ontology (GO) analysis, which organizes genes into hierarchical categories and uncovers gene regulatory networks on the basis of biological process and molecular function, was used to analyze the main function of differentially expressed genes (Gene Ontology, 2006). The KEGG pathway analysis was then used to identify the significant pathways for these genes (Kanehisa et al., 2004). The Database for Annotation, Visualization and Integrated Discovery (DAVID; https:/david.ncifcrf.gov/) provides a comprehensive set of functional annotation tools to analyze high-throughput gene function. GO and KEGG pathway enrichment analysis were performed using DAVID. We were only interested in biological processes, cell components, molecule functions, and KEGG pathways at the significant level (*p* < 0.05, FDR < 0.05, and an enrichment score of >1.5).

### Ingenuity Pathway Analysis (IPA)

The core pathway analysis was performed with Ingenuity Pathway Analysis (Andreas et al., 2014). IPA contains a curated database of networks and biological relationships based on original peer-reviewed articles. A geneset including DDX56 and closely related genes were uploaded and analyzed separately using the IPA software (Qiagen) (https://apps.ingenuity.com/).

### Western Blot Analysis

Cells were harvested in RIPA buffer. Protein concentration was measured using the BCA protein assay (HyClone-Pierce, Rockford, IL, USA). Equal amounts of total protein of each treatment were separated using 12.5% SDS-PAGE and further transferred onto PVDF membranes. Membranes were incubated with mouse anti-FLAG or anti-GAPDH antibodies (Santa Cruz Biotechnology, Santa Cruz, CA, USA). Secondary antibodies conjugated to horseradish peroxidase and ECL Western blotting reagents were used for detection.

### Quantitative Real–Time Polymerase Chain Reaction

Total RNA was extracted using the Trizol reagent (Invitrogen, Shanghai, China) and reverse transcribed to single-stranded cDNA. The cDNA was then used as a template for the following polymerase chain reaction (PCR). The primers used were as follows: for DDX56 forward, 5′-CCG CTT ATG CTA TTC CGA TGC-3′ and reverse, 5′-TGC GAG ATG GGG TCC CTA CTA TAG-3′; and for GAPDH forward, 5′-TGA CTT CAA CAG CGA CAC CCA-3′ and 5′-CAC CCT GTT GCT GTA GCC AAA-3′. GAPDH was used as an internal control. The PCR products of DDX56 and GAPDH were 258 and 121 bp, respectively. All samples were examined in triplicates.

### Recombinant Lentiviral Vector Production and Cell Infection

The interfering target sequence of DDX56 (ACTCAAGGAGCTGATATTA) was designed from the full-length DDX56 sequence (NM_019082) by GeneChem Co. Ltd. (Shanghai, China). After testing knockdown efficiencies, the stem-loop oligonucleotides were synthesized and inserted into the lentivirus-based pGCSIL-GFP (GeneChem Co. Ltd.) with AgeI/EcoRI sites. For lentivirus infection, U-2 OS cells were cultured into six-well plates, and then, the DDX56–shRNA–lentivirus or negative control (NC) lentivirus was added according to a multiplicity of infection (MOI). After 72 h of infection, the cells were observed under a fluorescence microscope (MicroPublisher 3.3RTV; Olympus, Tokyo, Japan). After 120 h of infection, the cells were harvested to determine knockdown efficiency by quantitative RT-PCR.

### Cell Growth Assay

Cell growth was measured using multiparametric high-content screening (HCS). Briefly, U-2 OS cells at the logarithmic phase after being infected with either the NC lentivirus or DDX56–shRNA lentivirus were seeded at 2,000 cells/well into 96-well plates; the cells were then incubated at 37°C with 5% CO_2_ for 5 days. The cells in the plates were counted using the Celigo® Image HCS Cytometer (Nexcelom Bioscience LLC, Lawrence, MA, USA) for each day's analysis. In each well, at least 800 cells were analyzed. Each experiment was performed in triplicate.

### *In vitro* Proliferation Assay

MTT assays were performed to measure the rate of cell proliferation *in vitro*. Briefly, the cells transfected with shDDX56 or shCtrl were planted into 96-well plates at a density of 1 × 10^5^ cells/well and then cultured for 24, 48, or 72 h, respectively. The transfected cells were incubated with 25 μl of MTT (Sigma-Aldrich) for 4 h at 37°C, followed by removing of supernatants and adding of 150 μl of DMSO (Sigma-Aldrich). The absorbance value was measured at 450 nm with a microplate reader (BioTek Instruments, Winooski, VT, USA).

### Cell Apoptosis Analysis

Flow cytometry (FCM) analysis was used to determine the cell cycle distribution or detect apoptosis. Briefly, U-2 OS cells were infected with DDX56–shRNA or NC plasmids and incubated at 37°C for 1, 2, 3, 4, or 5 days. At the indicated time point, adherent cells were collected. The suspension was filtered through a 300 mesh, and the DNA content of the stained nuclei was analyzed for the cell cycle phase by BD FACS Calibur flow cytometer (BD Biosciences, San Diego, CA, USA). Each experiment was performed in triplicate. Cell apoptosis was assayed by staining with Annexin V-APC (eBioscience, San Diego, CA, USA) and detected by FCM. For the analysis of apoptosis, U-2 OS cells were cultured into six-well plates. After 48 h of transfection with DDX56–shRNA or NC plasmids, the cells were collected and washed twice with ice-cold PBS. The cell concentrations were adjusted to 1 × 106/ml with 1× staining buffer. One hundred microliters of cell suspension was stained with 5 μl of Annexin V-APC at room temperature in the dark for 15 min. Cells were analyzed using FCM within 1 h. All experiments were performed in triplicate.

### Statistical Analysis

Statistical analysis was performed using SPSS for Windows version 23.0 (SPSS, Inc., Chicago, IL, USA). The Student's *t*-test was used for raw data analysis. The random variance model *t*-test was performed using BRB-ArrayTools (v4.6, http://linus.nci.nih.gov/BRB-ArrayTools.html) (Wright and Simon, 2003). The statistical data for each group were presented as the mean ± SD. Because the sample size was limited, the adjusted *p*-values were too large after multiple testing control. We used raw value of *p* < 0.05 as threshold for nominally significant differential expression. Notably, multiple testing adjustment with FDR < 0.05 was used to filtrate enriched GO and KEGG pathways.

## Results

### Identification and Preliminary Screening of DEGs

**Figure 1:**
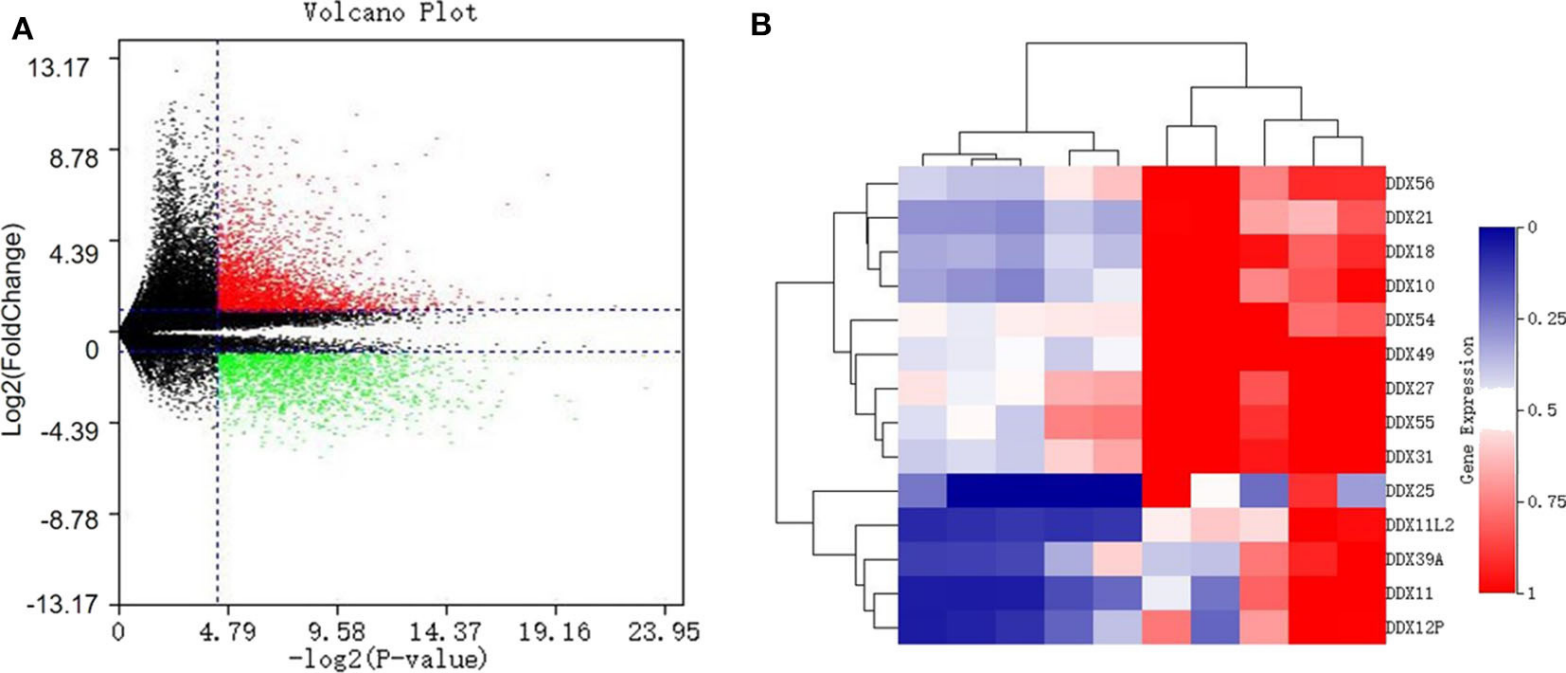
Identification of differentially expressed genes (DEGS) in the DEAD-box helicase (DDX) family between osteosarcoma tissue and adjacent normal tissue. **(A)** Volcano plot of all 60,492 expression genes included in the GSE126209 dataset. Red spots: up-regulated DEGs; green spots: down-regulated DEGs. **(B)** The heatmap with hierarchical clustering for differentially expressed genes in DDX family.

With a fold change (FC) cut-off value >2 and a value of *p* < 0.05, a total of 4,939 mRNAs (3,301 up-regulated and 1,637 down-regulated) were identified as differentially expressed between tumor and adjacent normal tissue from the gene expression microarray profile (Figure 1A). Genes (4,424) succeeded in the Ensembl Gene ID—gene symbol transferring and 515 genes failed. The 4,424 genes were then uploaded to the IID website for bone tissue-specific protein–protein interactions. A total of 32,292 PPIs were identified among those genes. We selected genes in DEAD-box (DDX) RNA helicases family for further screening (Table 1 and Figure 1B). The top rank gene DDX56 with highest node degree was chosen as the potential focal target.

**Table 1:** Protein–protein interaction network statistics of differentially expressed genes in DEAD-box helicase (DDX) family between osteosarcoma tissue and adjacent normal tissue.

**Rank**	**Gene symbol**	**Degree**	**Betweenness centrality**	**Closeness centrality**
1	DDX56	206	2.24E−04	0.371734
2	DDX54	193	3.29E−04	0.385697
3	DDX55	167	3.69E−04	0.363594
4	DDX5	141	2.07E−04	0.412069
5	DDX31	141	1.11E−03	0.362973
6	DDX6	140	4.36E−04	0.392576
7	DDX27	131	1.11E−04	0.373173
8	DDX11	124	1.70E−04	0.3568
9	DDX10	115	1.56E−04	0.369817
10	DDX3X	87	9.14E−05	0.402981
11	DDX1	80	5.91E−05	0.398811
12	DDX17	79	7.82E−05	0.398782
13	DDX20	69	3.69E−05	0.369504
14	DDX12P	66	4.81E−06	0.338923
15	DDX49	64	2.62E−05	0.35585

### mRNA Profile Data and Survival Analysis

**Figure 2:**
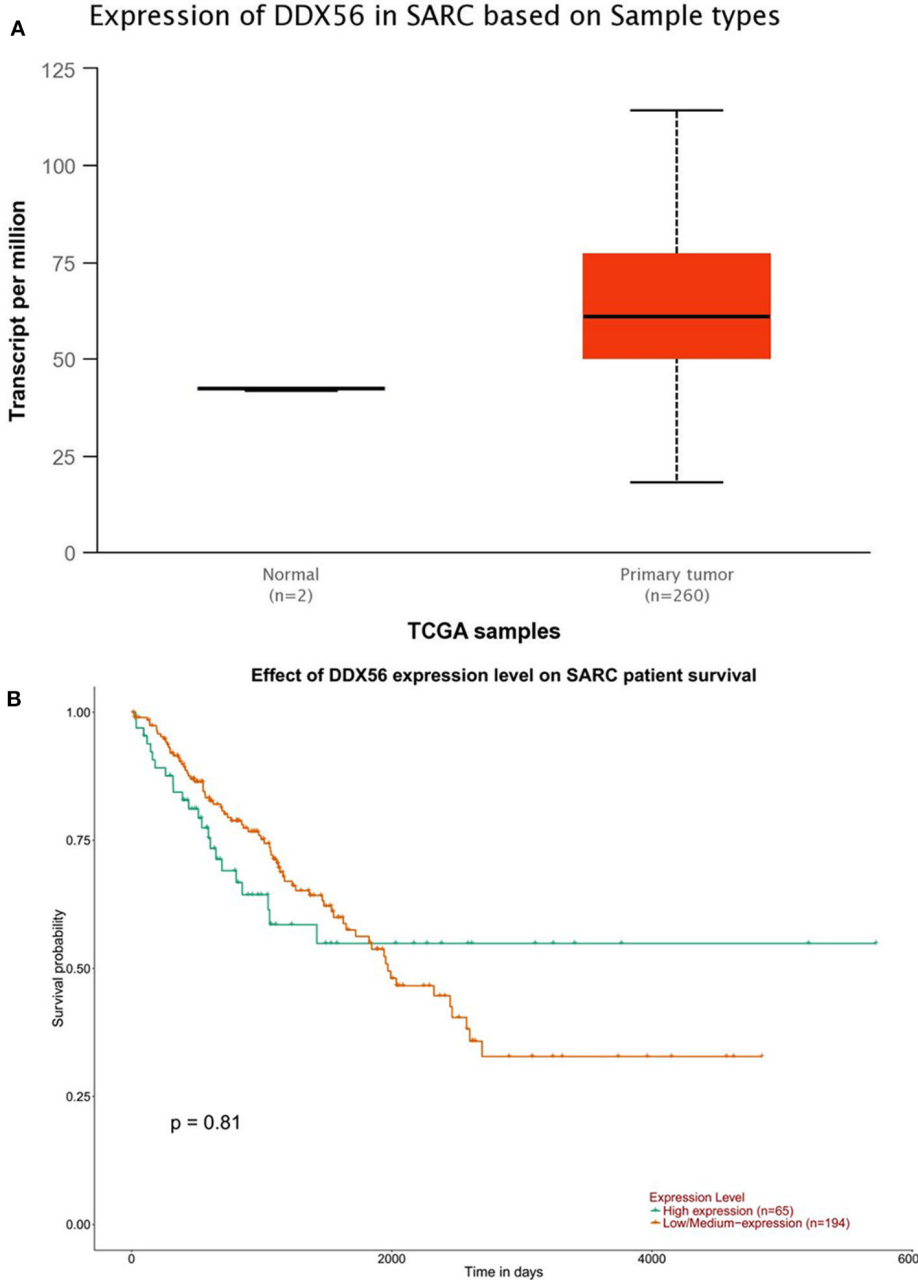
The Cancer Genome Atlas (TCGA) gene expression and survival plot of DDX56 in sarcoma patients. **(A)** The expression was significantly higher in sarcoma patients than in negative control (NC; *p* < 0.05). **(B)** Survival plot of DDX56 expression level and race on sarcoma (SARC) patient survival (*p* = 0.81).

**Figure 3:**
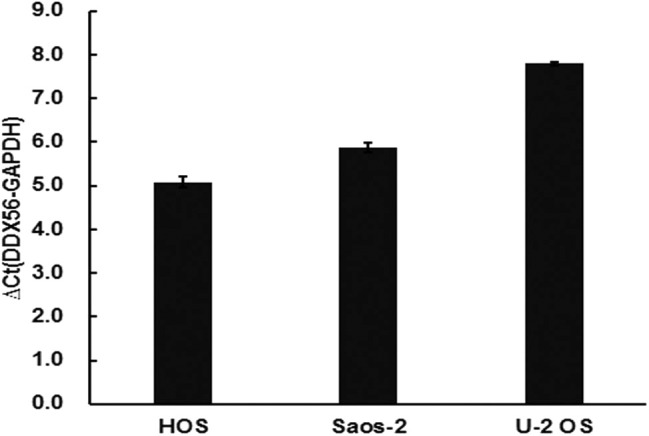
Validation of DDX56 expression levels in three osteosarcoma cell lines. Expression of DDX56 mRNA was measured by real-time qPCR in the indicated cell lines. A constitutively expressed GAPDH gene was used as an internal control (Ct, cycle threshold; ΔCt = Ct target gene – Ct internal control).

The TCGA data retrieved from UALCAN portal were used to analyze the gene DDX56 expression in SARC patients. These data revealed that when compared with normal tissues, DDX56 exhibited a significant higher expression level in tumor (*p* < 0.05) (Figure 2A). This difference is independent of gender and race (Figures S2A,B). These findings were consistent with the previous DDX56 expression analysis in GSE126209 dataset and *in vitro* validation in three different osteosarcoma cell lines. The gene expression of DDX56 is upregulated in HOS, Saos-2, and U-2 OS cell (Figure 3). Survival analysis was also performed to evaluate whether DDX56 expression levels could predict overall prognosis. However, using all of the TCGA data obtained, the Kaplan–Meier plot demonstrated no significant differences (*p* = 0.81) (Figure 2B). The patients with higher DDX56 expression had a low survival rate before 3 years of onset, while it was the opposite after 3 years. The crosspoint of two plots is near 1,926 days. Stratification was made, but no demographic bias was found (Figures S2C,D).

### Functional Enrichment Analysis

**Figure 4:**
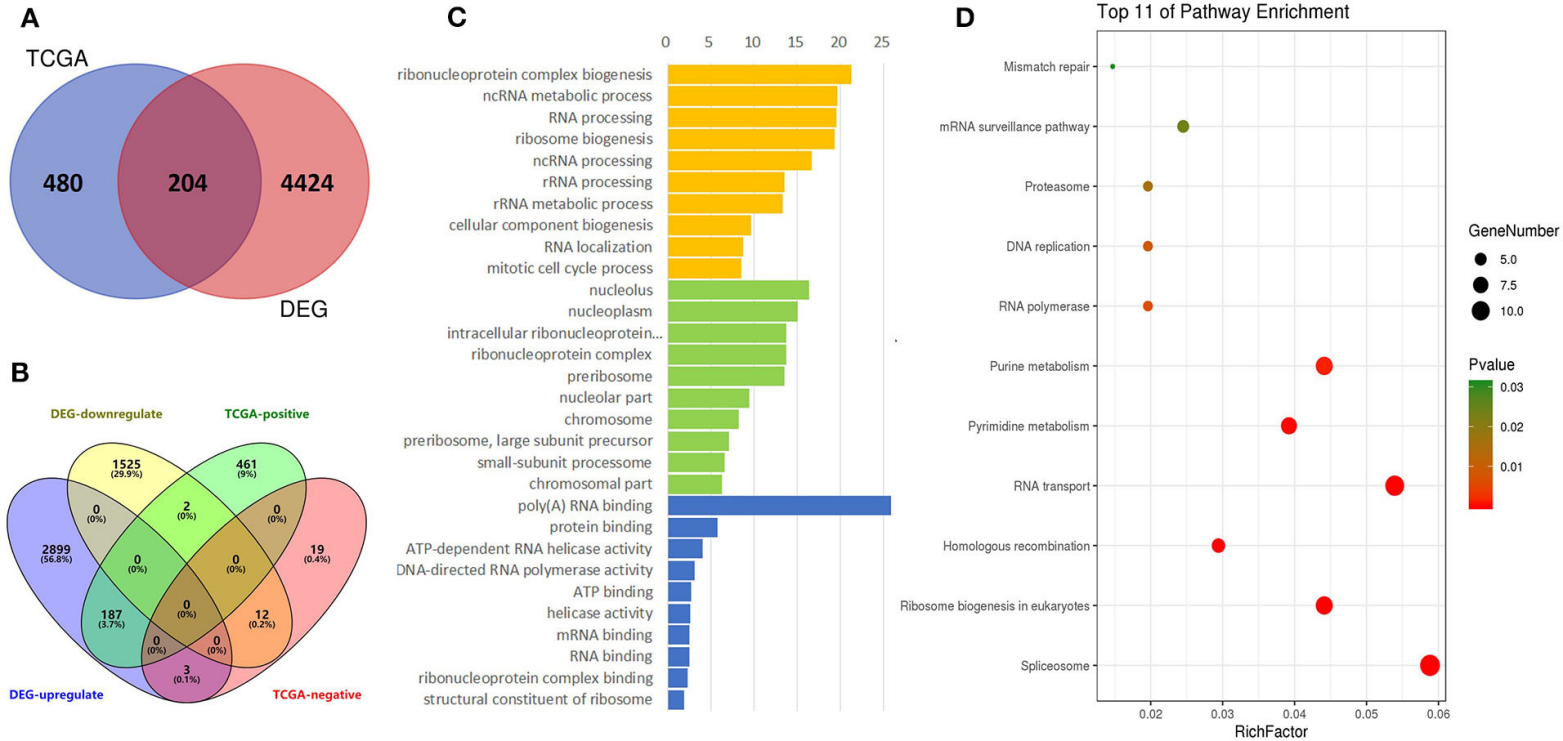
Identification of DEGs, gene ontologies (GOs), and KEGG pathways relevant to DDX56. **(A)** Venn diagram for selected identical DEGs and TCGA-relevant genes. **(B)** Four-dimensional Venn diagram for up/down-regulated DEGs and positive/negative-related TCGA genes. **(C)** Functional GO enrichment of DDX56-relevant DEGs. **(D)** KEGG pathway enrichment.

The TCGA data were also used to predict potential genes relevant to DDX56 function through the UALCAN portal. This prediction uses additional gene information base on GTEx profiles. This online *in silico* analysis yields a total of 480 potential genes relevant to DDX56 function. We found 204 genes co-expressing and interacting with DDX56 by intersecting these genes with the above DEGs (Figure 4A). More specifically, when the relationship with DDX56 was divided into positive and negative and the differential expression was divided into upregulated and downregulated, there were 199 genes in this intersection (Figure 4B). The GO analysis and KEGG analysis were performed using DAVID. The top enriched biological processes were ribonucleoprotein complex biogenesis, ncRNA metabolic process and RNA processing. The top enriched cell components were nucleolus, nucleoplasm, and intracellular ribonucleoprotein complex. The top enriched molecule functions were poly(A) RNA binding, protein binding, and ATP-dependent RNA helicase activity, respectively (Figure 4C). The most enriched pathways include spliceosome, ribosome biogenesis in eukaryotes, and homologous recombination (Figure 4D).

### Ingenuity Pathway Analysis

**Figure 5:**
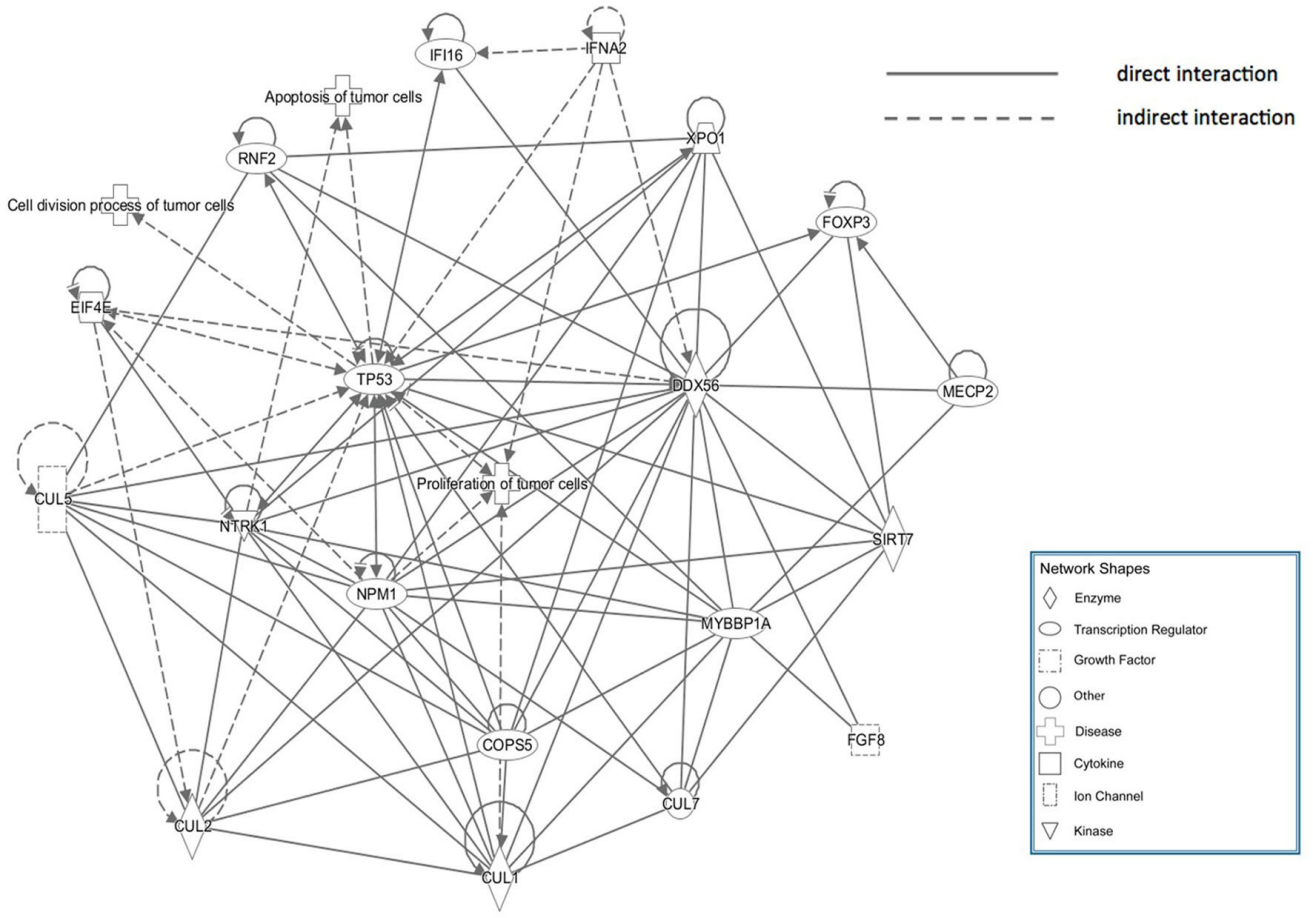
Ingenuity Pathway Analysis (IPA) network of genes that directly interact with DDX56, which were enriched in cell proliferation, apoptosis, and cell cycling.

The core pathway analysis was performed using the IPA software. The role of DDX56 in cell function includes pluripotency, replication, and growth. It has several mutations found in liver neoplasm, melanoma, and pancreatic ductal adenocarcinoma. Physical interactions, including RNA–RNA, protein–protein, protein–nucleic acid, and protein–cell or tissue were also found (Supplementary Material). A physical interaction network was built based on these findings (Figure 5). This manually curated database returned a network consisting of three diseases, eight transcription regulators, one growth factor, two cytokines, one ion channel, four enzymes, and several other integrities. The tp53 gene is a joint node in this IPA network, which has direct or indirect contacts with many other nodes, and participates in the communication and regulation of the entire access network. The nodes referencing relevant diseases in this network include proliferations, apoptosis, and cell division process of tumor cells.

### Lentivirus-Mediated Knockdown of DDX56

**Figure 6:**
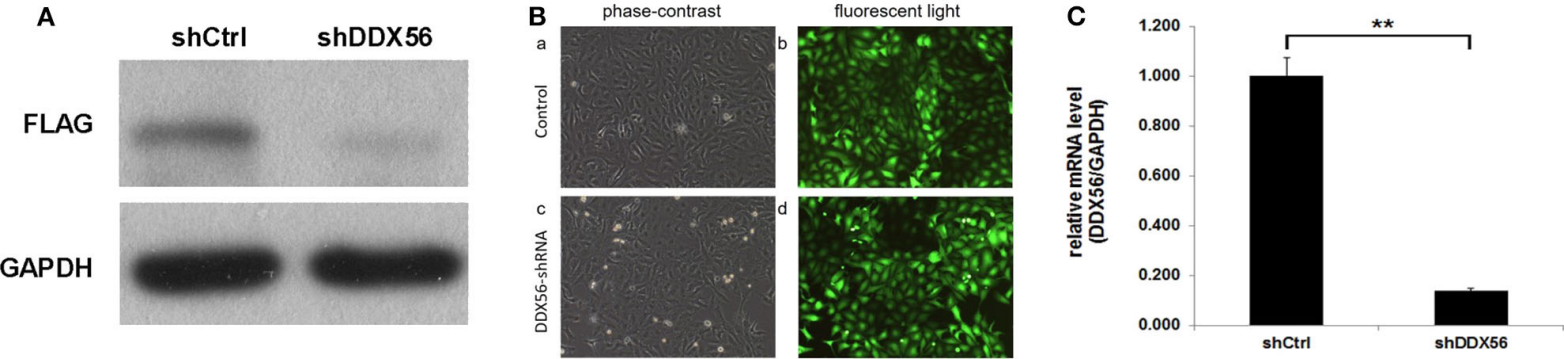
Knockdown of DDX56 protein expression in 293T cells. **(A)** External validation of DDX56 knockdown efficiency in 293T cells. DDX56 protein expression was analyzed by Western blotting in control-transfected (NC) and DDX56–shRNA-transfected 293T cells. GAPDH was used as a loading control. **(B)** Fluorescent microscopic images of U-2 OS cell lines infected with DDX56–shRNA and NC lentivirus vectors. Note that most of the cells express GFP. Magnification, ×100. **(C)** DDX56 mRNA expression was analyzed by real-time qPCR. Compared with shCtrl, DDX56 mRNA expression was markedly decreased after silencing by RNAi (shCtrl, sham shRNA interfered control cells; shDDX56, DDX56 targeted shRNA interfered U-2 OS cells; NC, normal control; **p < 0.01).

In order to validate the gene function of DDX56 in osteosarcoma, knockdown of the expression of DDX56 was performed by introducing a lentivirus cell infection model specifically designed. The knockdown efficiency was determined by external Western blot analysis using human embryonic kidney 293T cells. As is shown in Figure 6A, the target protein expression was detected by Western blotting in the cells, but was greatly reduced in the DDX56–shRNA-infected cultures, indicating effective knockdown of the target sequence. To further explore the role of DDX56, we knocked down DDX56 in the U-2 OS cell lines. As shown in Figure 6B, the proportion of infected cells was >80% for both the DDX56–shRNA and NC lentivirus by day 3 post-infection. DDX56 mRNA levels were assessed by real-time PCR at day 5 post-infection with either the DDX56–shRNA or NC lentivirus. The DDX56–shRNA lentivirus-infected cultures had significantly lower levels of DDX56 mRNA compared to levels in the cultures infected with the NC lentivirus (Figure 6C), which indicates the success of DDX56 knockdown in targeted cells.

### Knockdown of DDX56 in U-2 OS Cells Reduces Cell Proliferation

**Figure 7:**
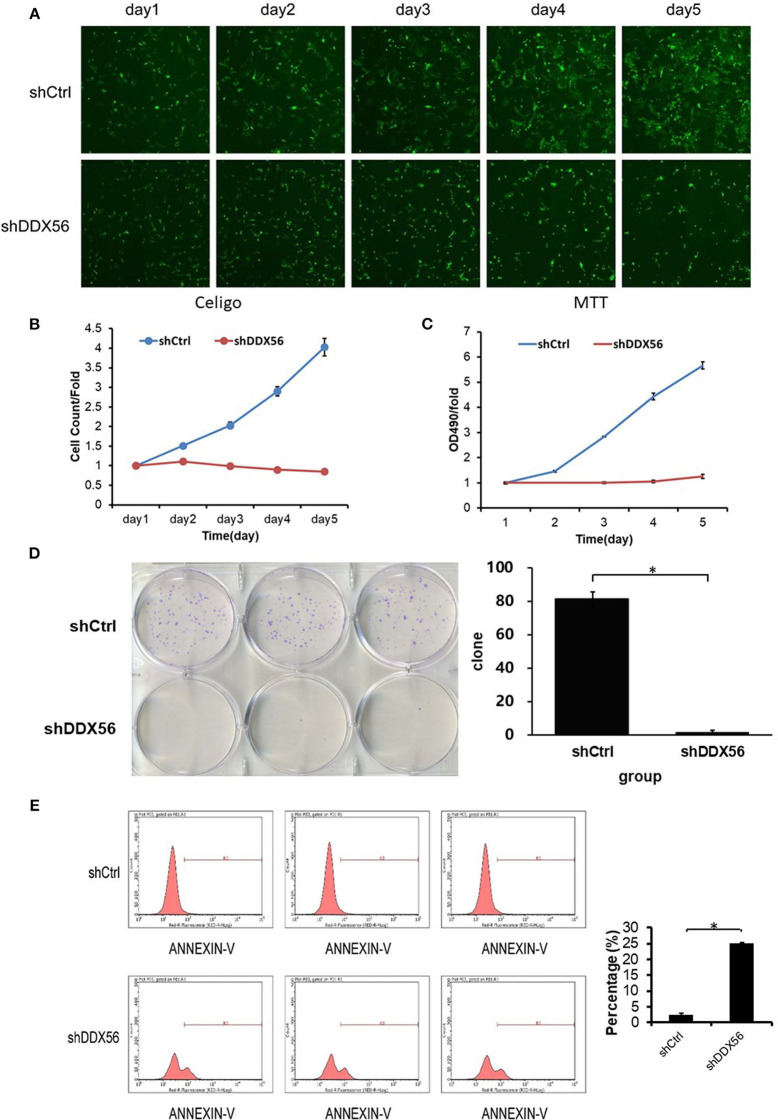
Effect of DDX56 knockdown on U-2 OS cell growth. **(A)** Cells were infected with the control or DDX56–shRNA lentivirus, and high-content cell imaging was applied every day as indicated to acquire raw images (unprocessed by software algorithm) of cell growth. **(B)** Cells were seeded into 96-well plates and infected with the control or DDX56–shRNA lentivirus, and cell growth was assayed every day for 5 days (NC vs. DDX56–shRNA, *p* < 0.05). **(C)** MTT measurement of cell proliferation was performed in cells infected with the control or DDX56–shRNA lentivirus. The number of viable cells was 2,000 per well. The optical density (OD) at 490 nm was recorded in 5 days (NC vs. DDX56–shRNA, *p* < 0.05). **(D)** Effect of DDX56 knockdown on U-2 OS cell clonogenic ability. Colony formation assay was performed. Cells were seeded into a six-well plate 3 days after lentivirus. The number of viable cells was 400. The picture (left) was captured 9 days after seeding using a digital camera. The statistics showed significant difference in clonogenic potential between groups (NC vs. DDX56–shRNA, *p < 0.05). **(E)** Effect of DDX56 knockdown on U-2 OS cell apoptosis. Cell death was determined by Annexin V staining and flow cytometry. Cell cultures showed a significant increase in apoptosis compared with NC (*p < 0.05).

To examine the effect of DDX56 on cell growth, U-2 OS cells cocultured with DDX56–shRNA or NC lentivirus were seeded into 96-well plates and were monitored by high-content screening (HCS) every day for 5 days. As illustrated in Figure 7A and confirmed by quantification in Figure 7B, control-transfected cells greatly expanded over the 5 days of the experiment, while the number of DDX56–shRNA-transfected cells did not change. The cell growth rate was defined as: Cell count at *n* days/cell count at first day, where *n* = 2, 3, 4, and 5 (Figures 7B,C). The results of the present study showed that DDX56 knockdown significantly inhibited cell growth rate of the U-2 OS cells.

### Knockdown of DDX56 in U-2 OS Cells Inhibit Clone Formation

In order to validate the cell clonogenic capacity change after DDX56 knockdown, MTT and clonogenic assays were used. Three days after shRNA lentivirus infection, the cells were plated in six-well plates; the number of plated cells was 400, and the number of clones was observed after 9 days. As is shown in Figure 7D, the results showed that the number of colonies in the experimental group decreased, suggesting that the DDX56 expression is closely related to the clonogenic capacity in U-2 OS cells.

### Knockdown of DDX56 in U-2 OS Cells Can Trigger Cell Apoptosis

The Annexin V–APC staining and flow cytometry analysis was carried out to test the relationship between cell apoptosis and DDX56 expression in U-2 OS cells. As is shown in Figure 7E, 4 days after shRNA lentivirus infection, U-2 OS cells in the experimental group showed a significant increase in apoptosis portion (shCtrl 2.53 ± 0.33% vs. shDDX56 25.05 ± 0.24%, *p* < 0.001). These results indicate that DDX56 expression is a determinant of cell apoptosis in U-2 OS cells.

## Discussion

Osteosarcoma is the most prevalent primary bone tumor in children, adolescents, and elderly adults (Mckenna et al., 1987). The current overall treatment efficiency and recurrence remain unsatisfactory as the molecular mechanisms underlying the pathogenesis have not been fully determined. Recent studies revealed the potential effects of RNA splicing, assembly, and adjustment on tumor genesis (Inoue et al., 2019; Shuai et al., 2019; Suzuki et al., 2019). The DDX RNA helicases family represents the largest family of RNA helicases to be involved in cellular metabolism (Cordin et al., 2006; Patrick and Paul, 2006; Patrick, 2008). In this study, we found 15 encoding genes in the DEAD-box helicase family among 4,939 differentially expressed mRNAs between osteosarcoma tumor and adjacent normal tissues. Since protein–protein interactions play essential roles in various biological progresses (Wang et al., 2019), we then mapped the PPI network using the above coding genes. DDX56, the node gene with the highest degree in the PPI network was selected for further study.

Interaction network analysis has been proven effective in assisting to understand the pathogenesis of complex diseases (Wang et al., 2020). In this study, the ingenuity pathway analysis was used to find out potential mechanism and core pathways that might relate DDX56 to tumor cell proliferations, cell division, and apoptosis. The IPA database manually screened existing knowledge from over 20 years' literature and provides sensitive and accurate predictions on molecular interactions. Based on the DDX56-related osteosarcoma IPA network, the TP53 gene exhibited a crucial role in the connection and regulation of these nodes. The tumor protein p53 (TP53), also known as p53, is the most frequently mutated human gene that regulates the tumor suppression processes (Wang et al., 2014; Wang and Sun, 2016). It controls cell cycle arrest and apoptosis induced by chemotherapeutic agents including doxorubicin, by activating Bax, p21, PUMA (p53 Upregulated Modulator of Apoptosis), and Noxa (Levine et al., 1991). Previous studies found that p53 suppresses osteosarcoma cell proliferation, metastasis, and angiogenesis through inhibition of the PI3K/AKT/mTOR pathway (Song et al., 2015). Activation of p53-dependent signaling pathway promotes apoptosis in osteosarcoma cells and enhances sensitivity of osteosarcoma to the chemotherapy (Yuan et al., 2007; Yang et al., 2012). In the present study, we performed corresponding experiments to validate the effects of lentivirus-mediated DDX56 knockdown on these processes. First, we detected the mRNA and protein expression levels of DDX56 in osteosarcoma using public data and RT-PCR assay. We showed that DDX56 was upregulated in the GSE126209 dataset, TCGA SARC patients, and validated using osteosarcoma cell lines. Then, we downregulated DDX56 in U-2 OS cell lines via transfection of shRNA plasmids. We evaluated the role of DDX56 knockdown in proliferation, cell division, and apoptosis of osteosarcoma cells. As expected, we observed that DDX56 knockdown exerted a significant inhibitory effect on proliferation and clonogenic capacity, while significantly promoting cell apoptosis in U-2 OS cells. These findings suggest that the DDX56-modulated oncogenesis and p53 signaling-related osteosarcoma neoplasia may share a common molecular pathway. Further studies regarding the underlining mechanisms are worth exploring.

Moreover, we found 204 genes co-expressing and interacting with DDX56 in the studied gene profile. These genes are primarily involved in RNA processing-related pathways, including spliceosome, ribosome biogenesis in eukaryotes, and homologous recombination. Notably, alternative RNA splicing is an essential process to yield proteomic diversity in human malignancies (Inoue et al., 2019; Shuai et al., 2019), especially including osteosarcoma (Ajiro et al., 2016). Several DDX family members were reported to play roles in alternative splicing (Linder and Jankowsky, 2011; Bourgeois et al., 2016). DDX5 and DDX17 contribute to tumor cell invasiveness by regulating alternative splicing of several DNA- and chromatin-binding factors (Peters and Doets, 2009). As DDX56 shares common structures with the DDX family members (Linder and Jankowsky, 2011), DDX56 may also change splicing by spliceosome assembly alteration. A recent study has verified that DDX56 cell promotes proliferation in colorectal cancer through alternative splicing tumor suppressor WEE1 (Voss et al., 2015). We found in our experiment that DDX56 knockdown exerted a significant inhibitory effect on proliferation and clonogenic capacity, while significantly promoting cell apoptosis in U-2 OS cells. However, the underlining mechanism remains to be further verified.

Survival analysis considering DDX56 expression on the prognosis of osteosarcoma was performed using data from the TCGA database. Oddly, these data do not support significant difference in clinical outcomes between patients with high- and low-expressed DDX56 probably due to the significant heterogeneity between samples. In practice, it was also recognized that the overall prognosis was poor before chemotherapy (Anderson, 2016). Moreover, the number of samples with osteosarcoma in the TCGA database is limited. Even though it is the largest samples volume currently available with clinical and expression data, there are only 65 primary sarcoma patients included with high DDX56 expression, given that osteosarcomas represent fewer than 1% of cancers overall. In the TCGA database, DDX56 is overexpressed in osteosarcoma among other cancers. External validation was performed *in vitro* using several osteosarcoma cell lines to suggest that DDX56 might be a novel oncogene in osteosarcoma. More rigid designed prospective clinical survival observation as well as mechanical studies should be performed in order to further validate this hypothesis.

There are some limitations that should be acknowledged. First of all, the sample size is relatively small due to the low overall incidence of the rare musculoskeletal malignancy. Second, the selected subjects all come from the Chinese Uyghur population. Given the potential ethnic specificity, the genetic background is possibly different between the Uyghur cells and purchased cells, which might affect the extension of the conclusion. Third, the IPA program builds its models by querying the known literature. Unknown interactions could not be discovered through this analysis, and thus, it is likely that there are highly relevant interactions that do not emerge in IPA. The next limitation is the fact that, since IPA queries only known associations and interactions, genes about which little or nothing is known about the function of their products cannot be identified as hubs using this method. In addition, the PPI network was constructed using a previous published database (IID) (Kotlyar et al., 2016); those unidentified but existing protein interaction relationships may have been missed. Given these limitations, the models generated here must be considered preliminary and incomplete. Other predictive tools, such as predictions of protein interactions based on molecular structure, specific groups, may be good cross-validations (Deng et al., 2020; Hu et al., 2020). Further studies should also be done to illustrate the underlying mechanisms.

In conclusion, we have identified DDX56 as a novel oncogene using bioinformatics tools and demonstrated that DDX56 was overexpressed in osteosarcoma tissues and cell lines. Furthermore, DDX56 knockdown inhibited cell proliferation and promoted cell apoptosis in osteosarcoma. These findings propose that DDX56 may be considered as a potential therapeutic target for the treatment of osteosarcoma.

## Data Availability Statement

The datasets analyzed in the present study are available in the Gene Expression Omnibus repository, https://www.ncbi.nlm.nih.gov/geo/query/acc.cgi?acc=GSE126209.

## Ethics Statement

All experiments were conducted on commercially supplied cell lines. Therefore, ethics approval and written informed consent was not required for this study.

## Author Contributions

XZ conceived the idea and designed the project. WH performed the data analysis. CZ and XZ wrote the paper. XZ, CZ, and NK revised the manuscript. YS provided the administrative support. CZ obtained the funding support. All authors contributed to the article and approved the submitted version.

## Conflict of Interest

The authors declare that the research was conducted in the absence of any commercial or financial relationships that could be construed as a potential conflict of interest.

## Supplementary Material

Figure S1 The heatmap with hierarchical clustering for all 4,940 differentially expressed genes (DEGs) in GSE126209 dataset.

Figure S2 **(A)** Gender difference on expression of DEAD-box helicase (DDX)56 was not significant (*p* > 0.05). **(B)** The expressions of DDX56 are indifferent among sarcoma patients from different races (*p* > 0.05). **(C)** Survival plot-effect of DDX56 expression level and gender on sarcoma (SARC) patient survival. **(D)** Survival plot-effect of DDX56 expression level and race on SARC patient survival.
