# Supplementary material for: Integrated Analysis of DEAD-Box Helicase 56: A Potential Oncogene in Osteosarcoma
Source: Front Bioeng Biotechnol. 2020 Jun 26;8:588. doi: 10.3389/fbioe.2020.00588 (PMC7332757; doi:10.3389/fbioe.2020.00588)
Supplement: Supplementary file 1 [file Data_Sheet_1.PDF]

IPA Gene View: DDX56 (Mammalian)

Provide Feedback | Live Sup

Review the categorized literature findings and database information for this node.

Summary Human Mouse Rat

Entrez Gene Name: DEAD-box helicase 56

Synonym(s): 2600001H07Rik, D11Ertd619e, DDX21, DDX26, DEAD (Asp-Glu-Ala-Asp) box polypeptide 56, DEAD-box helicase 56, DEAD-box p II/Gu

NCBI CDD Domains (Superfamilies / Multi-Domains): DEAD-like helicases superfamily, DEXDc, P-loop containing Nucleoside Triphosphate Hydrolases

Protein Functions / Functional Domains: ATP dependent RNA helicase, enzyme, RNA binding

Subcellular Location: cellular membrane, granular components, nuclear envelope, nucleoli, Nucleus

Canonical Pathway: --

Targeted By miRNA Functional Cluster: miR-1202 (and other miRNAs w/seed UGCCAGC), miR-1267 (miRNAs w/seed CUGUUGA), miR-143-5p (and other miRNAs w/seed 5p (miRNAs w/seed CCUGUGC), miR-210-3p (miRNAs w/seed UGUGCGU), miR-214-5p (miRNAs w/seed GCCUGUC), miR-3083-5p w/seed GGCUGGG), miR-3120-5p (miRNAs w/seed CUGUCUG), miR-337-3p (and other miRNAs w/seed CAGCUCC), miR-3612 (an GGAGGCA), miR-423-3p (miRNAs w/seed GCUCGGU), miR-4443 (miRNAs w/seed UGGAGGC), miR-4450 (and other miRNAs w/see (miRNAs w/seed AAGGGAC), miR-450b-3p (and other miRNAs w/seed UUGGGGA), miR-4540 (miRNAs w/seed UAGUCCU), miR-46 w/seed GGAGCUA), miR-4671-5p (miRNAs w/seed CCGAAGA), miR-4688 (and other miRNAs w/seed AGGGGCA), miR-4709-5p (m CAACAGU), miR-4778-5p (miRNAs w/seed AUUCUGU), miR-486-3p (and other miRNAs w/seed GGGGCAG), miR-5008-3p (and oth CUGUGCU), miR-5582-5p (miRNAs w/seed AGGCACA), miR-6078 (miRNAs w/seed CGCCUGA), miR-626 (and other miRNAs w/see 5p (miRNAs w/seed GGGCAGG), miR-6748-3p (miRNAs w/seed CCUGUCC), miR-6755-3p (miRNAs w/seed GUUGUCA), miR-6772- GGGUGUA), miR-6777-5p (and other miRNAs w/seed CGGGGAG), miR-6781-5p (miRNAs w/seed GGGCCGG), miR-6811-3p (and o GCCUGUG), miR-6857-5p (miRNAs w/seed UGGGGAU), miR-7064-3p (and other miRNAs w/seed AGGGCCC), miR-708-5p (and ot AGGAGCU), miR-7974 (miRNAs w/seed GGCUGUG)

Top findings from Ingenuity Knowledge Base (show all 206 categorized literature findings)

regulates: --

regulated by: macrophages, Zaire ebolavirus, IFNA2, RNase A, sirolimus, EIF4E

binds: NPM1, RPL14, RPL18A, FGF8, MECP2, RBM34, RPL30, DGCR8, NEUROG3, HIST1H1T, RPL37A, PPAN, MAGEB10, RPL18, GPATCH4

role in cell: pluripotency, replication in, growth

disease: peripheral arterial disease

Human ▾ Isoforms From RefSeq ▾

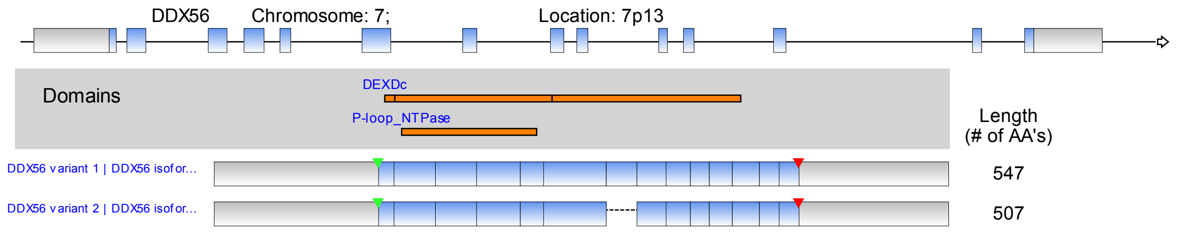

Descriptions from External Databases

Entrez Gene Summary: This gene encodes a member of the DEAD box protein family. DEAD box proteins, characterized by the conserved motif Asp-Glu putative RNA helicases. They are implicated in a number of cellular processes involving alteration of RNA secondary structure s initiation, nuclear and mitochondrial splicing, and ribosome and spliceosome assembly. Based on their distribution patterns, so family are believed to be involved in embryogenesis, spermatogenesis, and cellular growth and division. The protein encoded ATPase activity in the presence of polynucleotides and associates with nucleoplasmic 65S preribosomal particles. This gene ma ribosome synthesis, most likely during assembly of the large 60S ribosomal subunit. Multiple transcript variants encoding differ found for this gene. [provided by RefSeq, Mar 2012]

GO Annotations

Molecular Function: ATP binding; ATP-dependent RNA helicase activity; helicase activity; hydrolase activity; nucleic acid binding; nucleotide binding;

Biological Process: positive regulation of neuron projection development; ribosome biogenesis; RNA secondary structure unwinding; rRNA process

Cellular Component: cytoplasm; membrane; nucleolus; nucleus

2 Recently Added Findings (show Findings)

206 Categorized Literature Findings (hide details)

Functional Roles | Mutant Information | Modifications and Regulation | Disease | Expression and Localization | Physical Interactions

Functional Roles

Molecular Processes

secretion of (1) RNA

Cellular Processes

replication in (2) embryonic cell lines, epithelial cell lines, kidney cell lines

growth of (1) cells

pluripotency of (1) embryonic cell lines, embryonic stem cell lines, stem cell lines

Organismal Processes

replication of (2) West Nile Virus strain NY99

release of (1) West Nile Virus strain NY99

Mutant Information

somatic (4) liver neoplasm, melanoma, pancreatic ductal adenocarcinoma

silent mutation (3) liver neoplasm, melanoma

missense (2) adenocarcinoma, pancreatic ductal adenocarcinoma

heterozygous (1) adenocarcinoma

Modifications and Regulation

expression regulated by (41) IFNA2, Marburg virus, Reston ebolavirus, Zaire ebolavirus, activation, macrophages, miR-1202 (and other miRNAs w/seed UGCCAC
miR-1267 (miRNAs w/seed CUGUUGA), miR-143-5p (and other miRNAs w/seed GUGCAGU), miR-1914-5p (miRNAs w/seed CCUGUG
miR-210-3p (miRNAs w/seed UGUGCGU), miR-214-5p (miRNAs w/seed GCCUGUC), miR-3083-5p (and other miRNAs w/seed GGCUG
miR-3120-5p (miRNAs w/seed CUGUCUG), miR-337-3p (and other miRNAs w/seed CAGCUCC), miR-3612 (and other miRNAs w/see
miR-423-3p (miRNAs w/seed GCUCGGU), miR-4443 (miRNAs w/seed UGGAGGC), miR-4450 (and other miRNAs w/seed GGGGAUU),
miR-4475 (miRNAs w/seed AAGGGAC), miR-450b-3p (and other miRNAs w/seed UUGGGGA), miR-4540 (miRNAs w/seed UAGUCCU),
miR-4633-3p (and other miRNAs w/seed GGAGCUA), miR-4671-5p (miRNAs w/seed CCGAAGA), miR-4688 (and other miRNAs w/see
miR-4709-5p (miRNAs w/seed CAACAGU), miR-4778-5p (miRNAs w/seed AUUCUGU), miR-486-3p (and other miRNAs w/seed GGGG
miR-5008-3p (and other miRNAs w/seed CUGUGCU), miR-5582-5p (miRNAs w/seed AGGCACA), miR-6078 (miRNAs w/seed CGCCUG
miR-626 (and other miRNAs w/seed GCUGUCU), miR-6721-5p (miRNAs w/seed GGGCAGG), miR-6748-3p (miRNAs w/seed CCUGUC
miR-6755-3p (miRNAs w/seed GUUGUCA), miR-6772-5p (miRNAs w/seed GGGUGUA), miR-6777-5p (and other miRNAs w/seed CGG
miR-6781-5p (miRNAs w/seed GGGCCGG), miR-6811-3p (and other miRNAs w/seed GCCUGUG), miR-6857-5p (miRNAs w/seed UGG
miR-7064-3p (and other miRNAs w/seed AGGGCCC), miR-708-5p (and other miRNAs w/seed AGGAGCU), miR-7974 (miRNAs w/seed

localization regulated by (3) Dengue virus 2, Rubella virus

dissociation regulated by (1) RNase A

transcription regulated by (1) sirolimus

translation regulated by (1) EIF4E

Disease

Positive (1) peripheral arterial disease

Expression and Localization

Cell or Tissue Expression

Positive (15) breast cancer cell lines, cervical cancer cell lines, embryonic cell lines, fibroblast cell lines, hepatoma cell lines, keratinocyte cancer
skin cancer cell lines, smooth muscle cell lines, tumor cell lines

Negative (1) embryonic cell lines, fibroblast cell lines

Subcellular Location

Positive (6) granular components, nucleoli

Negative (1) cajal bodies

Physical Interactions

RNA-RNA

Positive (37) miR-1202 (and other miRNAs w/seed UGCCAGC), miR-1267 (miRNAs w/seed CUGUUGA), miR-143-5p (and other miRNAs w/seed GU
miR-1914-5p (miRNAs w/seed CCUGUGC), miR-210-3p (miRNAs w/seed UGUGCGU), miR-214-5p (miRNAs w/seed GCCUGUC),
miR-3083-5p (and other miRNAs w/seed GGCUGGG), miR-3120-5p (miRNAs w/seed CUGUCUG), miR-337-3p (and other miRNAs w/s
miR-3612 (and other miRNAs w/seed GGAGGCA), miR-423-3p (miRNAs w/seed GCUCGGU), miR-4443 (miRNAs w/seed UGGAGGC),
miR-4450 (and other miRNAs w/seed GGGGAUU), miR-4475 (miRNAs w/seed AAGGGAC), miR-450b-3p (and other miRNAs w/seed U
miR-4540 (miRNAs w/seed UAGUCCU), miR-4633-3p (and other miRNAs w/seed GGAGCUA), miR-4671-5p (miRNAs w/seed CCGAAG
miR-4688 (and other miRNAs w/seed AGGGGCA), miR-4709-5p (miRNAs w/seed CAACAGU), miR-4778-5p (miRNAs w/seed AUUCUG
miR-486-3p (and other miRNAs w/seed GGGGCAG), miR-5008-3p (and other miRNAs w/seed CUGUGCU), miR-5582-5p (miRNAs w/s
miR-6078 (miRNAs w/seed CGCCUGA), miR-626 (and other miRNAs w/seed GCUGUCU), miR-6721-5p (miRNAs w/seed GGGCAGG),
miR-6748-3p (miRNAs w/seed CCUGUCC), miR-6755-3p (miRNAs w/seed GUUGUCA), miR-6772-5p (miRNAs w/seed GGGUGUA),
miR-6777-5p (and other miRNAs w/seed CGGGGAG), miR-6781-5p (miRNAs w/seed GGGCCGG), miR-6811-3p (and other miRNAs w,
miR-6857-5p (miRNAs w/seed UGGGGAU), miR-7064-3p (and other miRNAs w/seed AGGGCCC), miR-708-5p (and other miRNAs w/s
miR-7974 (miRNAs w/seed GGCUGUG)

Protein-Protein

Positive (127) CAND1, CEP164, COPS5, CRB1, CUL1, CUL2, CUL4B, CUL5, CUL7, Capsid, DCUN1D1, DDX56, DGCR8, DHX37, DHX57, EED, EHD2, F
FURIN, GNL2, GPATCH4, HECTD1, HIST1H1T, HIST1H2BC, IFI16, MAGEB10, MECP2, MRPL43, MRPS14, MRPS24, MRPS25, MRPS34, M
NEK4, NEUROG3, NPM1, NS1, NTRK1, Ns5b, OBSL1, PPAN, RBM34, RNF2, RPGRIP1L, RPL14, RPL18, RPL18A, RPL23A, RPL26, RPL29
RPL34, RPL36A, RPL37, RPL37A, RPS11, RPS16, RPS29, RPS3A, RPS9, RRP12, SIRT7, SOX30, SPCS2, SRP14, SUZ12, TBC1D4, TTN, U
XPO1, ZNF408

Protein-Nucleic Acid

Negative (2) YBX2

Protein-Cell or Tissue

Positive (1) nucleoli

About Ingenuity | Contact Us

©2000-2018 QIAGEN. All rights reserved.
