# Supplementary material for: Integrated Analysis of DEAD-Box Helicase 56: A Potential Oncogene in Osteosarcoma
Source: Front Bioeng Biotechnol. 2020 Jun 26;8:588. doi: 10.3389/fbioe.2020.00588 (PMC7332757; doi:10.3389/fbioe.2020.00588)
Supplement: Supplementary file 2 [file Data_Sheet_2.PDF]

Preparation of target gene RNA interference lentiviral vector

Objective:

The DDX56 gene was used as a template to design RNA interference target sequences and construct target gene RNA interference lentiviral vectors. After completing the design of RNA interference targets, synthesize single-stranded DNA oligo containing interfering sequences, annealing and pairing to produce double-stranded DNA; then directly connect the enzyme-cut lentiviral vector through the restriction sites at both ends; The prepared E. coli competent cells were identified by PCR for positive recombinants and sent for sequencing verification. The sequencing results were compared to the correct clones for plasmid extraction.

Materials

Access Number： GV115

Component order： hU6-MCS-CMV-EGFP

Control insert： TTCTCCGAACGTGTCACGT

Tool carrier：

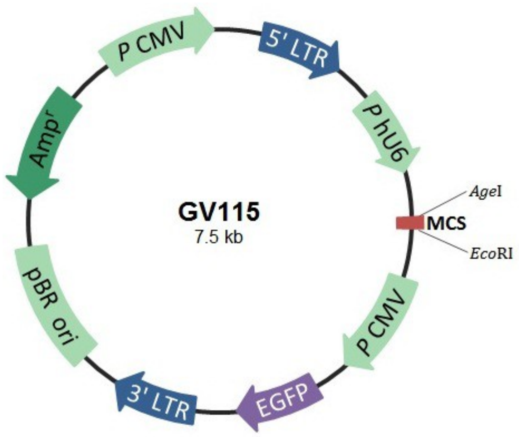

2. Experimental strains　TOP10 E. coli competent cells（TIANGEN, Cat. #CB104-03）

3. Experimental reagents

3.1 Enzyme reagents

Reagent	Supplier	Cat. #
Age I	NEB	R3552L
EcoRI	NEB	R3101L
CutSmart Buffer	NEB	B7204S

Taq Plus DNA Polymerase	Vazyme	P201-D3
T4 DNA Ligase	Fermentas	EL0016

3.2 Other reagents

Reagent	Supplier	Cat. #
dsDNA oligo	GENEray	
PCR primer（R&F）	GENEray	
TIANgel Midi Purification Kit	TIANGEN	DP209-03
EndoFree Maxi Plasmid Kit	TIANGEN	DP117
Tryptone	OXOID	LP0042
Yeast Extract	OXOID	LP0021
NaCl	Sangon Biotech	0241-500g
Tris	Sangon Biotech	T0826-500g
EDTA	Sangon Biotech	E0105-500g
Ampicillin，sodium salt	Genebase	A100339-0025
Agarose	SBS Genetech	GA4-100
250bp-II DNA Ladder	GENEray	DL2502
GeneRuler 1kb DNA Ladder	Thermo Scientific	SM0311
DNA sequencing	Meiji	ABI3733

3.3 Experimental Instrument

Instrument	Supplier	Cat. #
Digital display voltage regulator electrophoresis instrument	Tianneng	EPS200
Gel imager	Tianneng	Tanon-2500
Germ shaker	Hualida Lab Equipment Co.	HI-9211K
Water-isolated constant temperature incubator	Shanghai Yiheng Scientific Instruments Co.	GHP-9080
PCR amplifier	Applied Biosystems	2720 thermal cycler
Supercentrifuge	Thermo Scientific	Legend Micro 17
Nanodrop 2000	Thermo Scientific	
Gilson Pipette	Gilson	

4. Preparation of experimental reagents

4.1 Annealing buffer (pH=7.5-8.0)

Chemicals	Concentration
Tris	10mM
NaCl	50mM
EDTA	1mM

4.2 LB liquid medium（100ml，pH=7.0）

Chemicals	Weight	Mass percent
Yeast Extract	0.5g	0.5%
Tryptone	1g	1%
NaCl	1g	1%

* The amount of benzylpenicillin in LB medium containing benzylpenicillin was 100 μg/ml.

4.3 LB solid medium（100ml，pH=7.0）

Chemicals	Weight	Mass percent
Yeast Extract	0.5g	0.5%
Tryptone	1g	1%
NaCl	1g	1%
Agarose	1.5g	1.5%

*The amount of benzylpenicillin in LB medium containing benzylpenicillin was 100 μg/ml.

1. RNA interference target design and double-stranded DNA oligo preparation

Gene information

Symbol	Accession#	Species	Full name
DDX56	NM_019082	Homo sapiens	Homo sapiens DEAD-box helicase 56 (DDX56), transcript variant 1, mRNA

2. RNA interference target design

According to the RNA interference sequence design principle, multiple 19-21nt RNA interference target sequences were designed using the DDX56 gene as a template. The following sequences were selected as interference targets after evaluation measurements by the design software.

Target number	Internal number	target sequence	GC%
pSC-1	psc45728	ACTCAAGGAGCTGATATTA	36.8%

3.DNA oligo sequence synthesis

Design shRNA interference sequences based on the selected target sequences and add appropriate restriction endonuclease sites at both ends to complete vector construction. In addition, a TTTTT termination signal is added to the 3' end of the positive chain and a termination signal complementary sequence is added to the 5' end of the reverse chain. The design was completed and sent for synthesis of single-stranded DNA oligo.

Internal number	5' bases	STEM	Loop	STEM	3'bases
psc45728-1	CCGG	GCACTCAAGGAGCTGATATTA	CTCGAG	TAATATCAGCTCCTTGAGTGC	TTTTTG
psc45728-2	AATTCAAAAA	GCACTCAAGGAGCTGATATTA	CTCGAG	TAATATCAGCTCCTTGAGTGC	

*CCGG: AgeI restriction enzyme cutting site; AATTC: EcoRI restriction enzyme cutting site; G: EcoRI Complementary sequences at the enzyme site。

4. Double-stranded DNA oligo preparation

The synthesized single-stranded DNA oligo dry powder was dissolved in annealing buffer (final concentration 20 μM) in a water bath at 90°C for 15 min. After natural cooling to room temperature, a double-strand with sticky ends was formed.

Linearized carrier preparation

Prepare 50 μl reaction system according to the NEB instructions, and use AgeI and EcoRI to double digest GV115 vector to make it linear.

reagent	amount of usage
Vector (1 μg/μl)	2μl
CutSmart Buffer	5μl
AgeI (10 U/μl)	1μl
EcoRI (10 U/μl)	1μl
H_2O	Up to 50μl

Incubate at 37 ° C (optimum temperature) for 1 h, and then cut the gum to recover the target fragment.

Electrophoresis loading instructions

Lane 1: 1kb Marker: 10kb, 8kb, 6kb, 5kb, 4kb, 3.5kb, 3kb, 2.5kb, 2kb, 1.5kb, 1kb, 750bp, 500bp, 250bp in order from top to bottom

Lane 2: Vector plasmid after Age I and EcoR I double digestion linearization

Lane 3: Vector plasmid without enzyme digestion

Agarose gel electrophoresis pictures

RNA interference lentiviral vector construction

1. Connect

Prepare 20 μl reaction system according to the instructions of Fermentas T4 DNA Ligase, and connect the double-stranded DNA oligo to the linearized carrier.

reagent	amount of usage
Linearized Vector (100 ng/μl)	1μl
Insert (100 ng/μl)	1μl
10×T4 DNA ligase Buffer	2μl
T4 DNA ligase	1μl
H_2O	Up to 20μl

The reaction was carried out at 16 ° C for 1h-3h, the ligation product was named psc45728, and then the conversion experiment was carried out.

2. Transformation

Transform the ligation product into E. coli competent cells, the detailed steps are as follows:

1) Add 10 μl of ligation product psc45728 to 100 μl of E. coli competent cells, ice bath for 30 min.

2) Heat shock at 42 ° C for 90sec and ice bath for 2min.

3) Add 500 μL of antibiotic-free LB liquid medium, and shake at 37 ° C for 1 hr at 200 rpm.

4) Spread 150 μl of bacterial solution evenly on the LB solid medium containing Amp and incubate in a 37 ° C incubator overnight.

3. PCR identification of positive clones

3.1 RNA interference vector construction and positive clone identification diagram

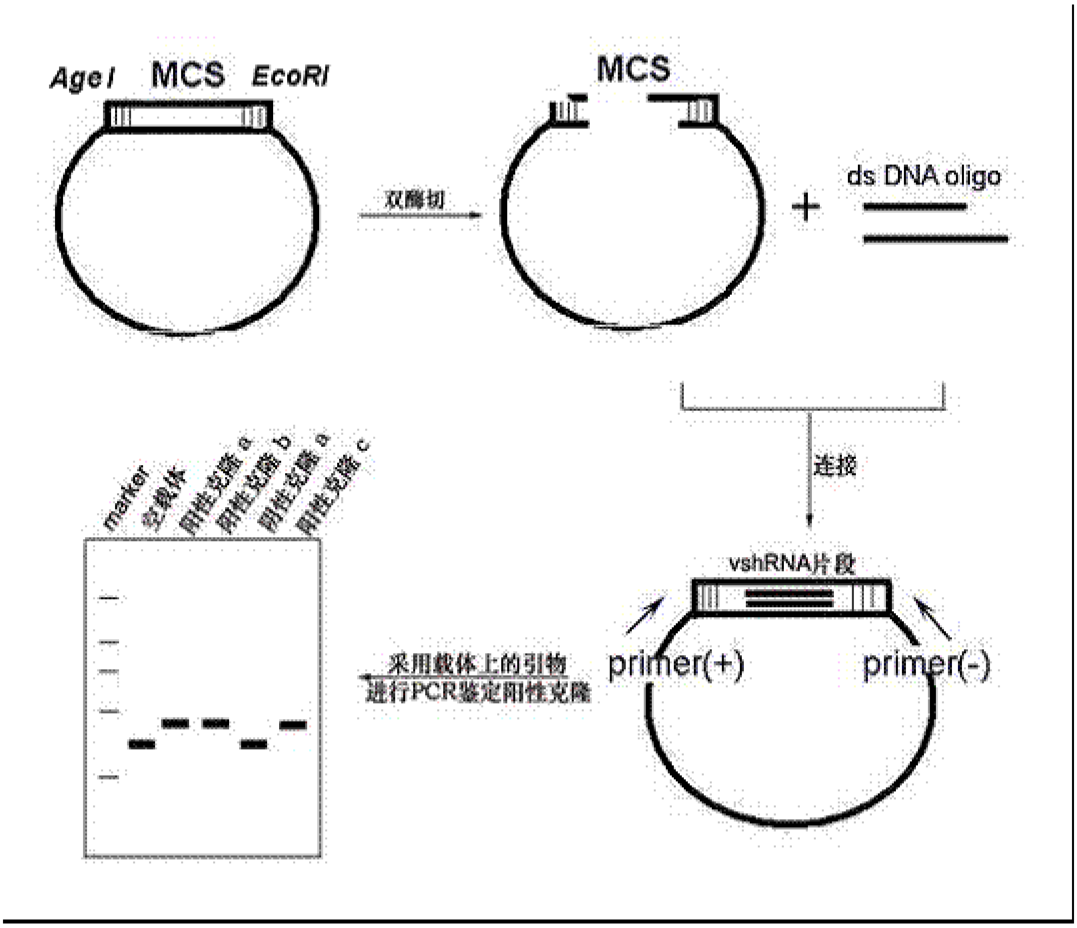

3.2 primer

Prime	Sequence（5'→ 3'）
Target primer -F	CCTATTTCCCATGATTCCTTCATA
Target primer -R	GTAATACGGTTATCCACGCG

3.2 PCR amplification

Prepare 20μl PCR reaction system according to the following table, use a sterile pipette to pick a single colony as a template for PCR amplification, the reaction conditions are: 94 ℃ 3min; 94 ℃ 30s, 55 ℃ 30s, 72 ℃ 30s, 22 cycles 72 ℃ 5min. After the PCR, 5 μl of the product was taken and the band was detected by 1% agarose gel electrophoresis.

Reagent	amount of
Taq Plus DNA Polymerase	0.2μl
10 x Buffer	2μl
Target primer -F	0.4μl
Target primer -R	0.4μl
template	-
H_2O	Up to 20μl

Electrophoresis loading instructions

Lane 1: Negative control (ddH2O), to exclude false positive results caused by foreign nucleic acid contamination in the system

Lane 2: Self-connected control (empty vector self-connected control group)

Lane 3: 250bp Marker: 5kb, 3kb, 2kb, 1.5kb, 1kb, 750bp, 500bp, 250bp, 100bp from top to bottom

Lanes 4-8: monoclonal psc45728-1,2,3,4,5

Agarose gel electrophoresis pictures

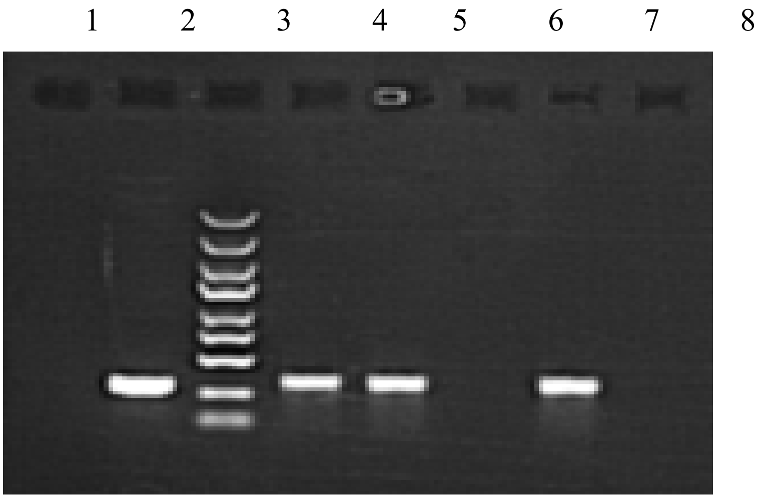

PCR band size

The size of the positive clone PCR fragment linked into the shRNA fragment is: 380bp;

The size of the PCR fragment of the empty vector clone without ligating into the shRNA fragment is: 307bp.

From this, psc45728-1,2,4 were judged as positive clones, and the clones with correct identification results were saved and sequenced.4. Analysis of sequencing results of positive clones.

Identify primer-F for positive clone sequencing, and select clones whose sequencing results are completely consistent with the target sequence for the next experiment.

psc45728 sequencing results

TTCTTGGGTAGTTTGCAGTTTTAAAATTATGTTTTAAAATGGACTATCATA
TGCTTACCGTAACTTGAAAGTATTTCGATTTCTTGGCTTTATATATCTTGTG
GAAAGGACGAAACACCGGGCACTCAAGGAGCTGATATTACTCGAGTAAT
ATCAGCTCCTTGAGTGCTTTTTGAATTCTCGACCTCGAGACAAATGGCAGT
ATTCATCCACGAATTCGGATCCATTAGGCGGCCGCGTGGATAACCGTATT
ACCGCCATGCATTAGTTATTAATAGTAATCAATTACGGGGTCATTAGTTCA
TAGCCCATATATGGAGTTCCGCGTTACATAACTTACGGTAAATGGCCCGC
CTGGCTGACCGCCCAACGACCCCCGCCCATTGACGTCAATAATGACGTAT

GTTCCCATAGTAACGCCAATAGGGACTTTCCATTGACGTCAATGGGTGGAGTATTTACGG

☆ The shRNA interfering sequence inserts are marked with red font, and the AgeI restriction site is destroyed.

Plasmid extraction

Transfer the correctly sequenced bacterial solution to 150 ml of LB liquid medium containing Amp antibiotics, and shake overnight at 37 ° C with shaking. Extract the plasmid according to the EndoFree Maxi Plasmid Kit instructions, and the qualified plasmid enters the downstream process.

The detailed operation steps are as follows:

1. Collect cells by centrifugation at 8000 rpm for 4 minutes.

2. Add 7ml P1, mix by shaking;

3. Add 7ml P3, mix upside down 6 ~ 8 times, resting for 5min;

4. Add 7ml P4, mix upside down 6-8 times, ice bath for 10min;

5. Centrifuge at 9000 rpm for 10 minutes, transfer the supernatant to the filter CS, add 10 ml of isopropanol after filtering and mix well;

6. Add 2.5ml of balance liquid BL to the adsorption column, centrifuge at 8000rpm for 2min, discard the waste liquid in the collection tube, and put the column back for use;

7. Pour the supernatant into the adsorption column twice, centrifuge at 8000rpm for 2min, and discard the waste solution;

8. Add 10ml of rinsing solution PW (absolute ethanol has been added) to the adsorption column, centrifuge at the same speed for 2min, discard the waste solution, and repeat this step once;

10. Add 3ml of absolute ethanol to the adsorption column, centrifuge at 8000rpm for 2min, and discard the waste liquid;

11. Spin at 9500 rpm for 5 minutes to remove residual rinse solution;

Transfer the adsorption column to a new white tube, add 800 μl of elution buffer TB (preheated first) dropwise at the center of the column, place at room temperature for 5 min, and then centrifuge at 9500 rpm for 2 min;

12. Transfer the eluent from the tube to a clean 1.5ml EP tube and store at -20 ℃;

13. Sampling electrophoresis, using a spectrophotometer (Thermo_Nanodrop 2000) to determine the plasmid concentration, quality inspection.

14. Transfer the qualified plasmid to the downstream platform for virus packaging.

Western Blot assay

Objective:

The Western Blot method was used to detect the protein expression level of the target gene knockdown in the cell, and then to determine the interference effect of the target.

Parameters

1. Gene information

Gene name number species

DDX56 NM_019082 people

2. Experimental cell lines

293T, adherent-dependent epithelioid cells.

3.Experimental antibody

3.1 Primary antibody

Antibody	Supplier	Product No.
Mouse anti-Flag	Sigma	F1804
Mouse anti-GAPDH	Santa-Cruz	sc-32233

3.2 Secondary antibody

Antibody	Supplier	Product No.
Goat Anti-Mouse IgG	Santa-Cruz	sc-2005

Western blot parameters:

SDS-PAGE separation gel concentration: 8%

loading volume: 20 µg

Color rendering system: ECL & X-ray film

Antibody dilution:

Primary antibody: Mouse Anti-Flag: 1:2000

Primary antibody: Mouse anti-GAPDH: 1:2000

Secondary antibody: Goat Anti-Mouse IgG: 1:2000

Lentivirus infection assay

Objectives:

In this experiment, the lentivirus containing the RNA interference sequence of the target gene was used for the target cell infection experiment.

Parameters:

1. Cell

Cell	Culture Environment	MOI	Infection	Plate	Cell Amount
U-2 OS	DMEM +10%FBS	10	Eni.S+polybrene	6-well	2×10^5

2. Virus

No.	Group	Concentration	Usage
psc3741	shCtrl	8×10^8 TU/ml	2.5 μl
LVpGCSIL-004PSC45728-1	shDDX56	5×10^8 TU/ml	4.0 μl
