# Supplementary figures and images for: Integrated Analysis of DEAD-Box Helicase 56: A Potential Oncogene in Osteosarcoma
Source: Front Bioeng Biotechnol. 2020 Jun 26;8:588. doi: 10.3389/fbioe.2020.00588 (PMC7332757; doi:10.3389/fbioe.2020.00588)

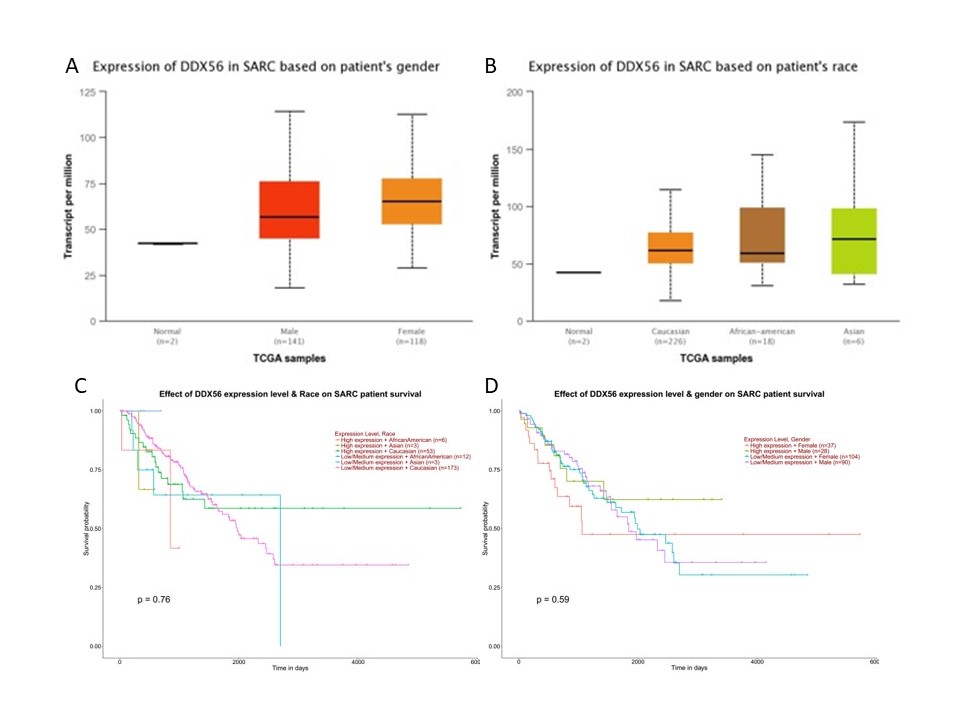

Supplement: Figure S2 — (A) Gender difference on expression of DEAD-box helicase (DDX)56 was not significant (p > 0.05). (B) The expressions of DDX56 are indifferent among sarcoma patients from different races (p > 0.05). (C) Survival plot-effect of DDX56 expression level and gender on sarcoma (SARC) patient survival. (D) Survival plot-effect of DDX56 expression level and race on SARC patient survival. [file Image_2.JPEG]
